# A Practical Treatise on Organic Diseases of the Uterus

**Published:** 1844-04

**Authors:** 


### Art. XV.

*A Practical Treatise on Organic Diseases of the Uterus.* By John C. W. Lever, m.d., Assistant Accoucheur at Guy's Hospital, &c. &c. —*London*, 1843. 8vo, pp. 240.

Dr. Lever's treatise is the substance of an essay on 'the Symptoms and Treatment of Organic Diseases of the Uterus,' to which was awarded the Fothergillian gold medal, by the council of the Medical Society of London, in 1843, and one of the reasons he assigns for its publication is, "that it has been the custom for some years past to submit to the public that Thesis which has been honoured with the Fothergillian Medal." The work is written in a very clear and unpretending style; and from the opportunities which our author possesses for the investigation of uterine diseases, (having from one to two hundred cases weekly under his inspection,) he must be well qualified to write upon the subjects of which it treats.

The volume is divided into three Parts. The First comprises inflammation of the uterus, acute and chronic, with their sequelæ; the Second, the specific diseases, under which term, he includes polypus, hard, fleshy or fibrous tumours and strumous tubercles, &c.; and the Third, the malignant diseases, as cauliflower excrescence, corroding ulcer of the uterus, melanosis and the varieties of cancer. The author commences his work with some statistical observations on functional and organic diseases of the uterus, the results of which show that functional disease is more frequent than organic in the proportion of 65·2 per cent. to 34·5 per cent. It results also that marriage favours the development of organic changes in the uterus: "of 95 females affected with polypus, carcinoma, fungoid disease, and hard tumours, 89 were married, and 6 were single." This is not to be wondered at, he says "considering the part the uterus has to perform, its turgescence during copulation, its increase of structure and development during pregnancy, and the accidents to which it is liable, (especially the mouth and neck,) during the process of parturition." (p. 5.) It appears that the diathesis accompanying organic disease does not impair the faculty of conception, since out of 89 married women, affected with various organic uterine diseases, 8 had never conceived, the child-bearing women amounting to 91·09 per cent. Dr. Lever also states that organic disease of the uterus does not materially affect the life of the child, and that when death does occur it appears to depend on the protraction of the labour from the non-dilatibility of the parts, and the consequent pressure upon the head; "81 women conceived 553 times, 43 conceptions, or 7·7 per cent., terminated in abortion, and 510 children, or 92·3 per cent., were born at the full period of utero-gestation. Of these 510 children, 12 only, or 2·35 per cent., were still-born." (p. 6.)

We pass over the general symptoms of organic disease of the uterus, and come to the first part of the work, in which Dr. Lever treats of inflammation of the uterus. This he divides into the acute and chronic, and treats of it as it occurs, 1, in the unimpregnated, and 2, in the impregnated uterus.

*Acute inflammation of the unimpregnated uterus* is not of frequent occurrence. It rarely shows itself before puberty, although Dr. Lever quotes a case from Dance, in which it was fully developed in a child eight years of age. It mostly arises from suppressed menstruation, or the use of strong injections for the cure of leucorrhea. We have seen a case produced by the irritation of menstruation. This form of the disease is rarely fatal, but Dr. Lever has seen three such cases. It most frequently terminates in resolution, sometimes it becomes chronic. Ramollissement is an occasional result of this disease, as well as abscess and gangrene, of which latter termination Dr. Lever does not speak from experience.

*Acute inflammation of the uterus during pregnancy and after labour* is next treated of, the latter being by far the most frequent during pregnancy. The inflammation may attack any part of the uterus, but most frequently that portion where the placenta is attached, producing adhesion of that body to the uterus. It may occur in the cervix of the uterus leading to ramollissement of the part. It is occasioned most commonly by cold, by mechanical injuries, as a fall, blow or attempts to induce premature labour by mechanical means, or the exhibition of medicines, especially ergot of rye. Inflammation occurring after labour may be caused by the injection of cold water or styptics, to allay hemorrhage, by violence when performing the operation of version or the unskilful use of instruments; we have seen it frequently arise without any assignable cause. Inflammation of the uterus during pregnancy may terminate in resolution, adhesion of the placenta to the uterus, occasionally in ramollisement, of which termination Dr. Lever relates a case which terminated fatally from laceration of the softened tissues. The substance of the case is as follows. H. A., a stout plethoric woman, twenty-five years of age, who had borne three dead children, was taken in labour with her fourth child on February 22, at half-past eight o'clock in the morning. Her medical attendant was sent for, and found the os uteri dilating. At half-past ten o'clock the os uteri was fully dilated, and the head presented in the second position. The pains were powerful and expulsive, but the head did not descend, and at half-past one o'clock Dr. Lever was sent for, but before he arrived symptoms of rupture of the uterus showed themselves. The woman complained of an unusual soreness of her right side; the pains ceased; vomiting with difficulty of breathing supervened, and there was great depression of the pulse. Dr. Lever immediately on his arrival delivered the woman by lessening the child's head, and the placenta immediately followed. She lived through the following day, but died early next morning. On making a post-mortem examination, a slit was discovered in the cervix uteri at the posterior and right side, forming a communication between the peritoneal sac and the vagina, about two inches in length; at this situation the uterine tissue was soft and easily lacerable.

Inflammation of the impregnated uterus may terminate in abscess and gangrene, which latter is very rare. Dr. Lever relates an interesting case of abscess of the uterus in a lady six months pregnant, which opened into the vagina. From an imprudent ride in a carriage, premature labour came on, and she expelled a dead child in a state of decomposition. Her convalescence was rapid, and in twelve months after, she gave birth to a second child without any untoward circumstances.

In the *treatment* of inflammation of the impregnated uterus, our author recommends local bleeding, general bleeding being seldom necessary. The system should be brought quickly under the influence of mercury and its action kept up for some time. Hip-baths and anodyne enemata are serviceable. After the acute symptoms have passed away, Dr. Lever recommends blisters, but prefers a repetition of them to the keeping one open. In the treatment of inflammation of the uterus after delivery, he bleeds locally and generally, according to the constitution of the patient, but is decidedly adverse to the administration of mercury. He says "so far as my experience has gone, mercury seldom appears to exercise any salutary influence over this disease; when it has been administered so as to affect the system rapidly, it seems to hasten the patient's dissolution, provoking diarrhea, &c., and I have long since omitted its employment." (p. 39.) He usually depends on general and local bleeding, anodyne fomentations, and the exhibition of diaphoretics, especially Dover's powder. He says "we not unfrequently find that women who have suffered from hysteritis do not again become pregnant. This will in most cases arise from closure of the Fallopian tubes, preventing the admission of the semen to the ovaries." (p. 39.)

*Chronic inflammation of the uterus* is usually a sequel of the acute form, but not always. Dr. Lever observes, that the improper use of the secale cornutum may give rise to this kind of inflammation, and that those who have suffered from this cause are not prone to reconceive, and also that "not unfrequently the pathological changes produced in the uterine tissues serve as a nidus for the deposition and subsequent development of malignant disease." (p. 44.)

Chronic inflammation may affect the whole organ, or be confined to the os and cervix of the uterus. The sequelæ of chronic inflammation are hypertrophy, engorgement, induration, simple ulceration and enlargement of the glands of the os and cervix uteri. These, with their treatment, are well detailed. We do not, however, quite like our author's definition of engorgement of the uterus, neither do we think it quite correct. He says, by "engorgement," I mean a varicose distended condition of the uterine vessels. The uterus when visually examined has a livid appearance." (p. 59.) A varicose condition of the vessels implies a morbid state of their coats. Does Dr. Lever mean to imply this? A varicose state of the vessels in other parts of the body is a very intractable disease, and yet the cases brought forward by our author to illustrate this form of the disease, were cured in a very short time, one in five weeks and the second in a month. We refer the reader to the cases themselves, which we should rather consider as examples of congestion of the uterus, in which the inflammatory symptoms had not perfectly subsided.

*The specific diseases of the uterus* include polypus, fibrous tumours, tubercles of the uterus connected with a strumous diathesis, syphilis and gonorrhœa uteri.

*Polypus.* Our author defines polypus uteri to be "a tumour generally round or oval attached to some part of the uterus by a neck or pedicle of smaller size than the body." (p. 80.) Polypi vary in size from a pea to a child's head; they are usually round or oval, but sometimes present the form of a gherkin. When seen by means of the speculum, their colour is found to vary: in some instances they are white, in others red, and occasionally livid and blue veins are sometimes observed traversing their surface. Dr. Lever divides polypi into three varieties: the fibrous, the cellular, (which is very rare,) and the glandular. These last are supposed to be morbid enlargements of the ovula nabothi.

Uterine polypi are most frequently developed in the cellular tissue connecting the muscular and mucous coat, but occasionally are found arising from the muscular coat itself, and derive an external covering from the mucous membrane of the uterus. Polypi may grow from any part of the uterus, from the fundus, body, cervix or os uteri; and it is of importance in a practical point of view to determine the seat of attachment. Persons of sedentary habits, and who reside in damp and unhealthy situations, and those of a lymphatic temperament, are more liable to polypi. According to Dr. Lever, he has found polypi more frequent in the unmarried than the married, in the ratio of 7 to 3.

One of the most prominent symptoms which attend this disease, is hemorrhage, which is not unfrequently mistaken for the menstrual secretion. Hemorrhages of this kind are irregular in their appearance, and also as to the quantity of blood discharged. As the disease advances, the discharge usually increases also. The discharge of blood does not appear to depend upon the size of the polypus, for we frequently find that with a small polypus as much blood is lost, (if not more,) as when the polypus is large. The menstrual periods are usually prolonged. In the intervals of the hemorrhage, there is usually more or less of other discharges; in some instances it is mucous (leucorrhea), in others it is of a watery nature, ill coloured and fetid. The patient complains of central pains with weight in the pelvis and bearing-down, tenesmus and difficulty in micturition. The frequent losses of blood produce an anemic condition of the constitution. There are vomitings, palpitation, œdema, and emaciation. According to Dr. Lever, uterine polypi are found as frequently sensible as insensible.

The diagnosis of polypus is extremely important, and to this accordingly our author devotes particular attention. It is liable to be mistaken for early pregnancy, prolapsus uteri, inversion of the uterus; scirrhous enlargement of the os uteri; the "vivaces" of Levret; cauliflower excrescence; fungoid polypoid growths of cancer; an elytrocele, and lastly, encysted tumours of the vagina, and tumours between the vagina and rectum. The most important accident which polypus is liable to be mistaken for is inversion of the uterus: "the history of the case, (our author says,) its origin at the time of delivery, together with the state of the vagina itself, the presence of the os uteri, &c., will assist us in forming our diagnosis," (p. 88,) and he might have added, an examination by the finger "per rectum," will discover the absence of the uterus. "In those cases where the inversion is of a chronic character, more difficulty is experienced in forming our opinion. Careful examination made by the finger or catheter will discover that the inverted uterus is encircled at its neck with a cul-de-sac of little or no depth; while in polypus the instrument may be carried some distance along its pedicle beyond the os uteri, through which it had passed." (p. 88.) These observations only apply to partial inversion of the uterus; when it is complete, the os uteri does not embrace the inverted uterus, but may be felt as a ring behind the cul-de-sac, formed by the vaginal membrane. Chronic inversion of the uterus may also be distinguished from polypus by bearing in mind, that the inverted uterus is large at first, but in process of time becomes smaller, whilst polypus, on the contrary, increases in size, and continues to increase so long as it is allowed to remain. "There is more pain and tenderness in the inverted womb than in polypus, which in some cases is but little sensible when stretched or pricked, although acutely sensible in others." (p. 88.)

With respect to the *treatment*, our author mentions four methods for the removal of polypi, viz., torsion, the ligature, excision, and the actual cautery. The operation by torsion is easily performed by grasping the polypus with the finger and thumb, or a pair of forceps, and twisting it gently round until the stalk breaks. Cellular polypi are the best adapted for this operation, and small fibrous polypi with thin and narrow pedicles. The most approved operation is that by ligature, and the instrument which our author uses is Gooch's canula, at the outer end of which a rack has been superadded, so that the ligature may be tightened from time to time by turning the rack. The ligature which Dr. Lever prefers is whipcord, he says "It has been shown by Mr. Walne, in the 'Medical Gazette' for July, 1836, that whipcord when moistened, increases in thickness, but diminishes in length; therefore such a ligature after its application, and when bathed in the discharges, will tighten itself very considerably." (p. 94.) Excision has been recommended by many authors. The only objection which our author has to it is the liability of subsequent hemorrhage. We may mention that an experienced surgeon of the present day recommends the twisting of the pedicle two or three times round before excising them, by this means, he says, the hemorrhage is prevented; this proceeding can only be followed in small polypi with thin necks. Of the actual cautery we know nothing as a remedy for polypus.

The cases which follow are interesting and illustrative of the practice recommended, and especially case xx, as showing under some circumstances the necessity and safety of applying the ligature during pregnancy. The case referred to is that of a woman four months advanced in pregnancy, who had been suffering from repeated losses from the vagina. When Dr. Lever saw her she was anemic from the loss of blood; on making an examination per vaginam, a polypus was discovered growing from the os uteri, of the size of a hen's egg. Such was the condition of the woman, that Dr. Lever feared further loss, and the ligature was applied. In seven days the polypus was detached; her health rapidly improved; she went her full time and was delivered of a living child.

In the case of polypus combined with pregnancy, our author considers that if the polypus do not interfere with the general health, or by its size will not obstruct delivery, the operation ought not to be performed till after delivery. If symptoms arise from its presence, or the tumour is too large to admit of delivery, its removal ought to be effected. When its removal is contemplated for the latter cause, excision must be the remedy, and Dr. Lever recommends the application of the ligature previous to excision, to prevent hemorrhage.

*Uterine tumours.* Under the term "hard, fleshy or fibrous tumours," Dr. Lever includes "those tumours of the uterus for the most part non-pediculated which are either non-malignant, or if malignant possess that characteristic in a very low degree." (p. 109.) They may arrive either from the subserous tissue or from the cellular tissue of the uterus itself, and sometimes they are generated beneath the mucous lining. They may be developed in any part of the uterus, but are more frequently found in the body and fundus. They vary in size from a pear to the pregnant womb, and are found of all forms. Those situated in the substance of the womb we have generally found to be of a rounded form, whilst those situated under the mucous tissue, frequently present a pyriform shape, project into the cavity of the uterus, and have a polypoid character. There is nothing known respecting the formation of these tumours. In most instances they arise without any assignable cause. At other times their origin has been attributed to a kick or blow, or coagulum of blood. These tumours are more frequently seen in unmarried and sterile women, though occasionally they complicate pregnancy.

The symptoms which attend these growths are in many instances so slight as not to attract attention until the tumour has attained a large size and produces pressure on the surrounding viscera. These symptoms will vary according to the situation of the tumour. If it be situated on the anterior part of the uterus, the bladder will be pressed upon and there will be frequent desire to make water; if on the posterior part of the uterus, the rectum will be encroached upon producing tenesmus, hemorrhoids, &c. In one instance which came under our notice, the pressure upon the rectum was so great as to require the introduction of a tube for the evacuation of the fecal matter. If the tumour be situated on the side, cramp and numbness of the inferior extremity will be present. Dr. Lever states that in one case in which the tumour was attached to the fundus, retroversion of the womb was produced, and the patient died from the effects of the displacement. Amenorrhea may or may not exist, and when the tumours arise from the submucous tissue, there are usually frequent attacks of hemorrhage which produce all the symptoms of anemia. The mammæ sometimes sympathise with the morbid changes going on in the uterus, and become enlarged.

These tumours are liable to be mistaken for pregnancy, congestion and induration of the womb, scirrhus, polypus of the uterus, ovarian disease, and various abdominal tumours; we refer the reader to the work itself for the various diagnostic marks.

Unless interfered with, these tumours may remain stationary for many years without interfering with the comfort of the patient, except that arising from their bulk. When, however, their size is such as to interfere with the functions of the bladder or rectum, death is sometimes occasioned by the mechanical obstruction. More frequently the tumours become inflamed from some accidental cause or from pregnancy; they soften in their centre, and destroy life by the constitutional excitement produced by the morbid changes in their substance. In regard to treatment, our remedial means have little influence over these hard tumours; and we quite agree with Dr. Lever, that the less done to them the better. To preserve the general health as good as possible, to avoid all causes likely to produce inflammation, and to meet the symptoms as they arise, are the principal indications of treatment. Sir C. Clarke mentions the spontaneous removal of these tumours; and our author says, "it may reasonably be asked, if such tumours are capable of spontaneous absorption, is it not probable they may be absorbed when the constitution is influenced by those remedies which are known to possess a peculiar and specific influence on that system?" (p. 119.) Dr. Ashwell believes, from his own experience, that iodine has some effect upon these tumours, and that the small hard tumours of the cervix may be "melted down and cured by iodine." Dr. Lever does not speak so sanguinely of this remedy, although he states he has seen a stop given to the growth of the tumour after the exhibition of iodine. We have tried iodine liberally, but without producing any sensible effect: it is, however, a remedy well worthy of further trial. When the tumour presents a polypoid character and projects through the os uteri, Dr. Lever recommends its removal by ligature or excision; but at the same time remarks that "the breadth of the base or uterine attachment is frequently so considerable that removal by ligature is seldom accomplished." (p. 121.) We are of opinion that it is our duty, in all cases where the tumour presents through the os uteri, and the ligature can be applied, to apply it, even though the portion to be removed be small; for experience has shown that the remaining portion will in time descend and project through the os uteri, and admit of the application of the ligature a second or third time, and thus the morbid structure may, in some instances, be removed as it were by instalments.

On the subject of pregnancy, associated with hard tumours of the uterus, Dr. Lever has made some very judicious and practical observations; and in the measures recommended by him for the relief of the patient we entirely coincide. We would only observe, that in those melancholy cases when the tumour obstructs the passage of the child at the time of labour, it is our duty to give full trial to the efforts of nature before artificial means are resorted to; for cases have occurred—and especially one related by Dr. Beattie in the 'Dublin Journal'—where the diameter of the pelvis was so diminished as to lead him to think that the Cæsarian section would be required; yet, after some time had elapsed, the tumour was drawn up by the action of the uterus, and allowed the child to be extracted.*

*Cauliflower excrescence and corroding ulcer of the uterus* are well detailed, and the description contains all that is at present known upon the subjects.

*Cancer of the uterus* is considered in a very elaborate article. The author gives the different varieties of it, and states the opinions of various writers on its pathology. He acknowledges the hereditary character of the disease, but does not believe that it can be communicated by infection or inoculation. From tables here given it is shown to occur very frequently: out of every seven cases of uterine disease, cancer of the uterus existed once. With regard to the exciting cause, he does not think that syphilis and violence will produce the disease, unless the cancerous diathesis exists. The results of his tables lead him to the opinion that unmarried women are less liable to be attacked with this disease than married women; for, out of "120 cases of carcinoma, unmarried women bore a proportion of 5·83 per cent., married women 8·66 per cent., and widows 7·5 per cent.; and the same tables show that the disease which

* See Dublin Journal, July, 1840, p. 411.

most frequently precedes cancer is congestive dysmenorrhea. The symptoms of cancer in its several stages are well described, and also its diagnosis. With regard to the treatment of cancer, our author does not advance anything new. He is very partial to the administration of arsenic in the first and second stage of the disease, and he says he "has reason to be satisfied with its 'modus agendi.'" We are glad to find that Dr. Lever does not speak favorably of the French practice of applying caustics to the ulcerated surface. It is of very little use to remove the surface whilst the parts underneath are affected with the disease. As to the radical cure of the disease by complete or partial excision of the uterus, we have but one opinion. Our author devotes about twelve pages and a half of his work to the consideration of this subject and does not speak in favour of it. The cases which have been recorded of the extirpation of the whole uterus do not place the operation in a very favorable light; most of the subjects of them have died within a short time after the operation, some in a few hours; and in those cases where the operation has been supposed to be successful the disease has reappeared and carried off the patients. There can be no doubt that the uterus may be removed with impunity; but the question is, can the cancerous uterus be extirpated with a prospect of success? All accounts of the operation speak to the contrary; nor, indeed, is it likely it should be otherwise. The operation is never recommended until ulceration has taken place, at which time the system is already contaminated by the disease. In this case, although the local ravages of the affection may appear to be limited to the uterus, yet it is impossible to speak positively upon this subject. It is of little use to remove the local affection whilst the morbid diathesis remains behind. The same objections apply to the excision of the os uteri in this disease. The difficulty of determining the exact limits of the disease, and the unwillingness of the patient to consent to so severe an operation until all the ordinary remedies have been tried in vain, (when the legitimate period for its performance will have passed away,) are such serious objections as ought to deter every surgeon from performing it. Excision of the os uteri has been recommended in cauliflower excrescence, and our author quotes a successful case of Dr. Simpson of Edinburgh. The woman rapidly recovered from the operation, and in a short time became pregnant, and was delivered in due time without any untoward circumstances. We may add from authority, that this patient has been again pregnant and delivered, and was, at our last report, in good health.

We have thus gone through the principal diseases treated of by our author; and although we have met with nothing very original, we have been, nevertheless, much pleased by the perusal of the work. Besides materials from other sources, it contains the results of the author's own experience, and is fully illustrated by cases which have come under his own immediate observation. It is written with great clearness and simplicity, and the concise but sufficiently full details of the various diseases of which it treats interest without wearying the reader. Every part of the volume exhibits good sound sense and correct judgment. The junior practitioner will find it a safe and sufficient guide to enable him to understand the various diseases of the uterus, and, so far as our present knowledge admits, to conduct the treatment of them successfully.

